# Complete genome sequence of mycobacteriophage Hashim76, a B13 subcluster phage, from Hanahan, South Carolina

**DOI:** 10.1128/mra.00409-25

**Published:** 2025-07-21

**Authors:** Riley M. Polic, Isabella E. Gustafson, Hams A. Kamil, Dhruvi B. Patel, Abigail C. Davis, Kennedy A. Gohl, Maxim J. Halma, Hannah G. Korper, Hannah S. Linde, Stephanie B. Moore, Mikayla G. Sonnenfeld, Nathan E. Thompson, Ryan P. Ware, Reese M. Ziegler, Mouna S. DiBenedetto, Christine A. Byrum

**Affiliations:** 1 Department of Biology, College of Charleston 2343, Charleston, South Carolina, USA; Portland State University, Portland, Oregon, USA

**Keywords:** mycobacteriophage, annotation, subcluster B13, actinobacteriophage, Hashim76, genome, SEA-PHAGES, virus, cluster B, siphovirus

## Abstract

Hashim76, a B13 subcluster bacteriophage extracted from mud (Hanahan, South Carolina), infects *Mycobacterium smegmatis* mc^2^155 and exhibits siphovirus morphology. It has a circular double-stranded DNA genome (70,230 bp; GC content = 70.1) containing 101 putative protein-coding genes but no tRNA sequences. Whole-genome BLASTn alignment revealed high similarity to Zenteno07 (93.91% identity, 90% query coverage).

## ANNOUNCEMENT

As interest in treating mycobacterial infections with bacteriophage therapy has increased (1, 2), there has been a broad effort to isolate and sequence relevant viruses (3). Currently, 2,624 mycobacteriophage genomes have been sequenced (https://phagesdb.org/) and the viruses categorized into clusters/subclusters based on sequence similarity, with those in the same cluster sharing >50% nucleotide sequence coverage and/or >35% gene content similarity (GCS) (4–6). Hashim76 (Family Bclasvirinae) is of particular interest as a member of the understudied B13 subcluster.

**Fig 1:**
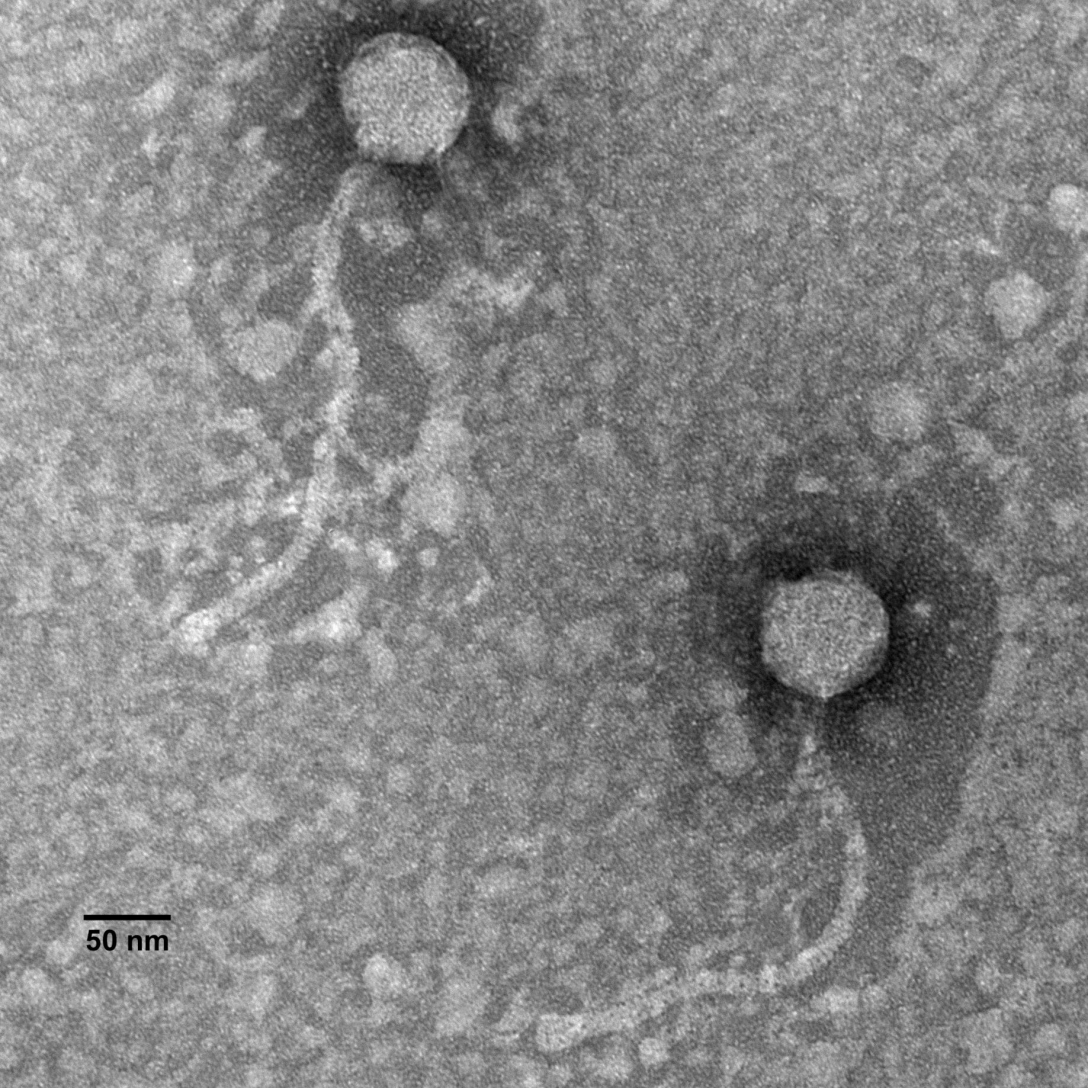
Morphology of the subcluster B13 bacteriophage Hashim76 examined using a JEOL 1010 transmission electron microscope at 80 kV. High-titer lysates collected on Formvar-coated copper grids were negatively stained with 1% uranyl acetate (4). Scale bar, 50 nm.

Hashim76 was isolated in September 2023 from mud collected beside a pond in Hanahan, South Carolina (32.95276 N, 80.03173 W). Soil surface samples (15 mL) were placed in a 50 mL conical tube, and 7H9 broth containing 1 mM CaCl_2_ was added to 35 mL. This sample was then washed for 1 h (250 rpm, 37°C), centrifuged (2,000 × *g*, 10 min), and filtered (0.22 µm pore). During enrichment, filtrates were inoculated with *Mycobacterium smegmatis* mc^2^155 (0.5 mL bacteria/25 mL filtrate), agitated for 3 days (250 rpm, 37°C), filtered again, plated using the double-layer agar method with 7H9 agar containing *M. smegmatis,* and incubated at 37°C (SEA-PHAGES Discovery Guide provides details) (7). After two purification/amplification cycles, plates densely populated with plaques were flooded with phage buffer, incubated for 2–4 h at room temperature, and lysate collected. Examination of Hashim76 by transmission electron microscopy also revealed siphovirus morphology with mean capsid diameter = 73.5 nm (*n* = 22), tail length = 309.9 nm (*n* = 21), and tail width = 12.6 nm (*n* = 22) (Fig. 1).

To study the genome, DNA was extracted from lysates (8.5 × 10^7^ plaque-forming units/mL) using the Promega Wizard DNA cleanup system, and the DNA library was prepared using an NEBNext Ultra(II) DNA Library Prep kit. Sequencing was performed on an Illumina MiSeq system (MiSeq reagent kit v.3) (8), and the 472,851 single-end reads (150 bp) were assembled into a single contig using Newbler v.2.9 (9). Further editing and finishing were performed on Consed v.29.0 (10), and because there was even coverage across the genome with no selective accumulation of reads, the genome is circularly permuted. For assembly/quality control details, see reference 8.

Genome annotation was performed using the PECAAN (11) workflow tool, and final files were transferred to DNA Master v.5.23.2 (https://phagesdb.org/DNAMaster). Putative genes were identified with Glimmer v.3.02 (12), Starterator v.1.1 (13), Genemark v.3.25 (14), Phamerator Actino_prophage v.5 (15), ARAGORN v.1.2.38 (16), and tRNAscan-SE v.3.0 (17). Domains and functional assignments were detected using BLASTp v.2.8.2+ (18), HHpred v.2.1 (19), TMHMM Deep v.1.0.42 (20), SOSUI v.1.11 (21), and the NCBI Conserved Domain Database (22). Default settings were applied to all programs except as summarized in https://seaphages.org/forums/topic/5398.

The Hashim76 genome is 70,230 bp long (955× coverage, 70.1% GC content) and contains 101 predicted protein-coding genes (Table 1). Thirty-one have identified putative functions, and four are unique to Hashim76 (gp66, gp84, gp87, and gp101). Five are unique to B13 subcluster viruses, occurring in all three subcluster members (gp62, gp76, gp82, gp96, and gp97), and no tRNAs are present. Based on whole-genome comparisons using BLASTn (18) and GCS (6), the closest viral relatives to Hashim76 are the other two B13 subcluster members, Zenteno07 from Round Rock, Texas (Genbank ON392167.1; 93.91% identity, 90% coverage, and 84.1% GCS) and BirdsNest from Kruger National Park, Phalaborwa, South Africa (Genbank MN813686.1; 90.76% identity, 84% coverage, and 78.7% GCS).

**TABLE 1:** Characteristics of the Hashim76 bacteriophage^*a*^

Parameter	Hashim76 data
GenBank accession no.	PQ844485
SRA accession no.	SRX27257323
Collection site	Hanahan, South Carolina, USA
Collection site coordinates	32.95276 N, 80.03173 W
Isolation host	*Mycobacterium smegmatis* mc^2^155
Genome size (bp)	70,230
Coverage (×)	955
GC content (%)	70.1
No. of predicted protein-coding genes	101
No. of tRNAs	0
No. of tmRNAs	0
Morphotype	Siphovirus
Subcluster	B13

^*a*^ A pham is defined as a potentially homologous protein-coding gene sharing ≥32.5% amino acid identity in CLUSTALW with BLASTp values <10^−50^ (15). Predicted phams unique to and conserved in all B13 subcluster members: gp62, gp76 (DNA-binding protein), gp82, gp96, and gp97.

## Data Availability

Hashim76 is available at the Pittsburgh Bacteriophage Institute in freezer box 163/grid H6. GenBank and SRA accession numbers are listed in Table 1.
